# Utilization of Self-Diagnosis Health Chatbots in Real-World Settings: Case Study

**DOI:** 10.2196/19928

**Published:** 2021-01-06

**Authors:** Xiangmin Fan, Daren Chao, Zhan Zhang, Dakuo Wang, Xiaohua Li, Feng Tian

**Affiliations:** 1 The Institute of Software Chinese Academy of Sciences Beijing China; 2 Department of Computer Science University of Toronto Toronto, ON Canada; 3 School of Computer Science and Information Systems Pace University New York, NY United States; 4 IBM Research Cambridge, MA United States; 5 Zuoshouyisheng Inc Beijing China

**Keywords:** self-diagnosis, chatbot, conversational agent, human–artificial intelligence interaction, artificial intelligence, diagnosis, case study, eHealth, real world, user experience

## Abstract

**Background:**

Artificial intelligence (AI)-driven chatbots are increasingly being used in health care, but most chatbots are designed for a specific population and evaluated in controlled settings. There is little research documenting how health consumers (eg, patients and caregivers) use chatbots for self-diagnosis purposes in real-world scenarios.

**Objective:**

The aim of this research was to understand how health chatbots are used in a real-world context, what issues and barriers exist in their usage, and how the user experience of this novel technology can be improved.

**Methods:**

We employed a data-driven approach to analyze the system log of a widely deployed self-diagnosis chatbot in China. Our data set consisted of 47,684 consultation sessions initiated by 16,519 users over 6 months. The log data included a variety of information, including users’ nonidentifiable demographic information, consultation details, diagnostic reports, and user feedback. We conducted both statistical analysis and content analysis on this heterogeneous data set.

**Results:**

The chatbot users spanned all age groups, including middle-aged and older adults. Users consulted the chatbot on a wide range of medical conditions, including those that often entail considerable privacy and social stigma issues. Furthermore, we distilled 2 prominent issues in the use of the chatbot: (1) a considerable number of users dropped out in the middle of their consultation sessions, and (2) some users pretended to have health concerns and used the chatbot for nontherapeutic purposes. Finally, we identified a set of user concerns regarding the use of the chatbot, including insufficient actionable information and perceived inaccurate diagnostic suggestions.

**Conclusions:**

Although health chatbots are considered to be convenient tools for enhancing patient-centered care, there are issues and barriers impeding the optimal use of this novel technology. Designers and developers should employ user-centered approaches to address the issues and user concerns to achieve the best uptake and utilization. We conclude the paper by discussing several design implications, including making the chatbots more informative, easy-to-use, and trustworthy, as well as improving the onboarding experience to enhance user engagement.

## Introduction

### Background

Medical consultation has historically been conducted between patients and their physicians during clinical encounters. However, some barriers may hinder effective communication in a clinical setting. For example, patients and their caregivers often face great challenges obtaining timely medical advice and information from health care providers due to the long wait time for an appointment [1,2]. The increasing demand for health care services also places a large burden on health care providers due to the shortage of medical professionals [3,4]. Medical professionals, therefore, have to overcome a range of temporal, geographical, and organizational barriers to provide a high quality of health care to patients [5]. Even more concerning is that health care infrastructures (the underlying foundation that supports the operations of a health care system) are complex and fragmented in many countries [6]. The scarceness and imbalanced distribution of health care resources (eg, facilities and medical professionals) often impede people in rural areas from accessing health care services and obtaining professional medical advice in a timely and effective manner [7,8]. Therefore, patients often use other mechanisms, such as online health forums and “Ask the Doctor” services, to seek medical help by asking questions and receiving answers from peers and health professionals who are willing to share their knowledge and opinions [1,9-12]. Even so, patients may still not be able to get instant responses using these online platforms [13,14]. Furthermore, there is much inaccurate information online, which may easily mislead patients [15].

With the advances in artificial intelligence (AI) technology in recent years, there is an opportunity to tackle the challenges and barriers faced by patients in seeking timely health information and to reduce the burdens posed on medical professionals [16,17]. That is, AI-driven intelligent systems, such as health chatbots, have emerged to support patients seeking medical advice irrespective of time and place [18]. These chatbots can provide live feedback to help patients get an overview of their symptoms, become aware of their illness, triage and manage their conditions, and ultimately improve their health [19-22]. Such chatbots act as a virtual conversational agent mimicking human interactions and offering medical advice (eg, diagnostic suggestions) directly to patients in a timely and cost-effective manner. In this way, health chatbots provide a form of triage into the health care system and become the first point of contact for health. While this technology is still in its developmental phase, chatbot systems could potentially alter the landscape of health care by increasing access to health care services, enhancing patient-centered care, and reducing unnecessary clinical visits [23,24].

Despite these potential benefits, similar to many other mobile health (mHealth) applications, chatbot systems have been inadequately adopted by those who might benefit most from this novel technology [4,25]. It is therefore important to examine how to design health chatbots to increase user adoption and engagement. Furthermore, prior work has focused primarily on developing advanced algorithms to improve the accuracy and effectiveness of chatbots’ diagnoses [21,26,27]. Few studies, however, have focused on the use of health chatbots in the real world [25]. More specifically, little is known about the “who, how often, and why” of chatbot use, what barriers and issues exist in using this novel technology, and how to overcome the barriers. This research gap is caused partly by the lack of large-scale deployment of health chatbots [28]. That is, most studies only examined the use of health chatbots in controlled settings rather than in real-world settings, where users interact with chatbots on their own.

To bridge this knowledge gap, we examined one widely deployed chatbot in China—DoctorBot [29]—which had attracted hundreds of thousands of users by the time this study took place. The large-scale deployment of DoctorBot provides us with a unique opportunity to gain an in-depth, empirical understanding of how people use health chatbots in the real world and what barriers hinder the delivery of these novel services. To the best of our knowledge, this is the first study that examined these issues using large-scale, real usage data. More specifically, we took a data-driven approach to analyze the system log of DoctorBot, which consisted of 47,684 consultation sessions initiated by 16,519 users between September 2018 and March 2019. Through this study, we make three contributions. First, we present a detailed analysis of how people use an AI-driven self-diagnosis chatbot, which remains understudied in the health informatics community. Second, we report issues and barriers that hinder the effective use of health chatbots. Third, our results can shed light on how to better design health chatbots to optimize user experience and achieve the best uptake and utilization.

### Features of DoctorBot

DoctorBot is an AI-driven, mobile-based medical consultation platform [29]. It utilizes large data sets (eg, numerous medical literature and clinical cases) and state-of-the-art AI techniques (eg, deep learning and knowledge graphs) to process users’ inquiries and provide personalized medical advice. Users can interact with DoctorBot by typing information into a chatbox and/or recording a voice message to express their health concerns (the voice message can be converted into text in real time). DoctorBot provides different health services to users, such as self-diagnosis, drug use instructions, diet suggestions, and so forth. Among those, self-diagnosis is one of the most popular and demanding services. Users can explain their health concerns to the chatbot and receive medical advice (eg, diagnostic suggestions and treatment options) to make informed decisions. Given the current heated debate on the readiness and usefulness of self-diagnosis chatbots [30,31], we chose to focus on the use of the self-diagnosis feature in this study.

**Figure 1:**
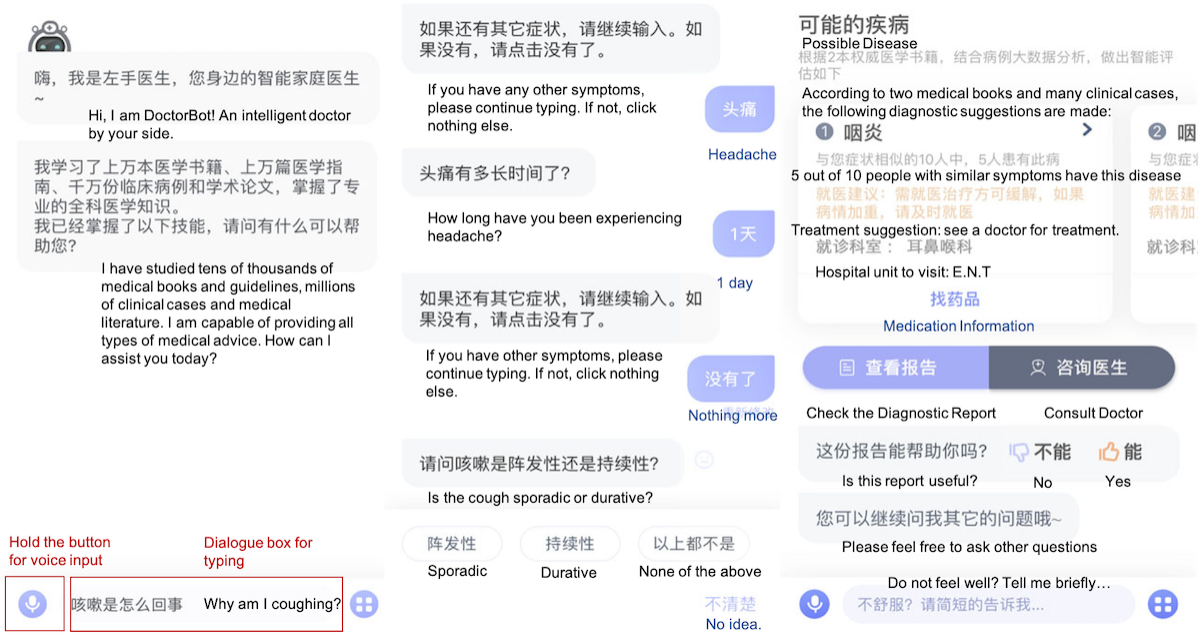
DoctorBot interfaces (all Chinese dialogue was translated into English in this figure). Left: the starting point of a consultation; after greetings, the user is prompted to explain health concerns. Middle: DoctorBot asks the user a series of symptom-related questions; users can select from the offered options at the bottom to provide an answer. Right: a report detailing potential diagnoses (ranked by their possibility) and other medical advice is provided to the user. E.N.T: ear, nose, and throat clinic.

A typical consultation session starts with a prompt for the user to describe their main symptom or chief complaint (Figure 1, left). After being prompted, the user provides input, such as, “Why am I coughing?” This inquiry triggers DoctorBot to take the initiative and ask the user a series of questions related to the symptoms (eg, “How long has the cough been present?”). The chatbot may also inquire if the user is experiencing other relevant symptoms (Figure 1, middle). For example, if a user indicates that he/she has been coughing, the chatbot would consecutively ask if the user is also experiencing a sore throat, shortness of breath, and so forth. If the answer is “yes,” the chatbot then asks the user more questions about each relevant symptom. Finally, DoctorBot asks the user to provide his/her medical history to conclude the consultation.

When a consultation is complete, DoctorBot generates a report detailing potential diagnoses, prediction confidence, treatment options, and which hospital unit (eg, cardiology, urology) to visit (Figure 1, right). Such information is expected to help users decide when, where, and whether or not to seek further medical help. It is also worth noting that DoctorBot explicitly instructs users to use the diagnosis for reference only, in light of AI liability issues and medical ethics [32].

## Methods

### Data Collection and Processing

To understand the use of self-diagnosis chatbots in the real world, we took a data-driven approach to analyze the system log of DoctorBot collected between September 2018 and March 2019. The data set consisted of 47,684 consultation sessions initiated by 16,519 users over the 6-month period. The log of each consultation session included a session ID, the user’s general information (eg, age, gender), consultation details (eg, time stamps, and conversation content between DoctorBot and the user), a diagnostic report automatically generated by the chatbot after the consultation was completed, and user feedback (if the user voluntarily provided feedback regarding the usefulness of the chatbot at the end).

The first author worked closely with the chatbot company to determine the type, amount, and format of usage data that needed to be extracted to fulfill the study’s purpose. To protect users’ privacy, all identifiable information (eg, phone number) was removed from the data set by the chatbot company before sending it to the researchers. Therefore, the data we analyzed was completely anonymized. Moreover, the users consented at the point of registration that researchers were allowed to analyze their usage data for research purposes. The study procedure was approved by the first author’s university’s Institutional Review Board.

The initial data set had some noisy data; for example, one consultation session could be stored as two separate sessions, and two consultation sessions could be merged. Therefore, we preprocessed the data by splitting the sticky conversations and spliced the broken conversations. After data processing, the research team examined the entire data set to ensure the accuracy and appropriateness of the data format. Since the study context was based in China, the content of the dialogues between the chatbot and users was in Chinese. To ensure the validity of the data, we decided to analyze the content in its original language. We used the Jieba [33] word segmentation library to segment the user input to extract semantic information.

### Data Analysis

To understand who uses chatbots, how often, and why, we first performed descriptive statistical analysis on the entire data set, focusing on user characteristics (eg, gender and age) and general patterns of chatbot use (eg, duration, frequency, and purpose of use). This analysis helped us not only to obtain a general overview of chatbot use but also to identify an interesting scenario—only 30,710 of 47,684 consultation sessions were completed, with the remaining 16,974 sessions being terminated by users before the chatbot reached a diagnosis. Therefore, we decided to split the entire data set into “completed” and “uncompleted” consultation sessions for further analysis. In particular, we used content analysis [34] and statistical analysis in combination to analyze both types of sessions to investigate the issues and barriers that may occur during the interactions between DoctorBot and users. More specifically, because an uncompleted consultation session may suggest that a user encountered barriers in using the chatbot, we investigated the exact moment when a consultation was terminated to understand what questions were asked by DoctorBot and how much time users spent on answering these questions. We also performed statistical analysis (ie, logistic regression and principal component analysis [PCA]) to explore the characteristics of uncompleted consultations. Examining these aspects could help us gain a preliminary understanding of the factors that could potentially lead to user dropout. In addition to the analysis of uncompleted sessions, two researchers performed a content analysis on 3000 completed sessions that were randomly selected to examine interaction issues that may exist in the use of the chatbot, such as miscommunication between DoctorBot and users. The content analysis was augmented by statistical analysis to further explore the influencing factors on the emerged interaction issues. Finally, as DoctorBot usually prompts the user to provide feedback (eg, positive versus negative experience) toward the end of the consultation, we performed a content analysis on the user feedback to identify user concerns and issues related to the use of DoctorBot and explored what factors could contribute or lead to these user concerns.

## Results

### General Patterns of Chatbot Use

We first report the results that emerged from the statistical analysis on the entire data set. This analysis helped us understand several patterns of the large-scale use of DoctorBot, including who used it, the length of each consultation, how often users used the application, and what health concerns users had queried about.

#### Who Used DoctorBot?

**Figure 2:**
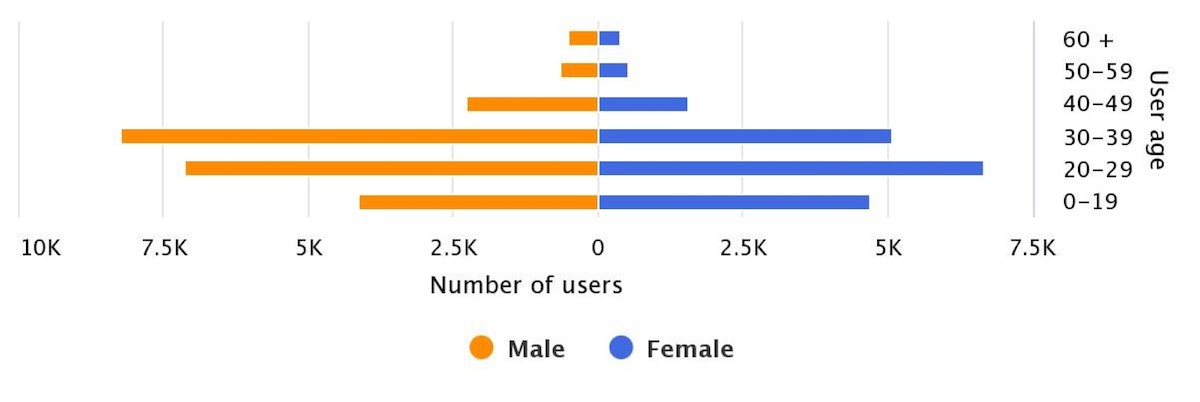
Characteristics of DoctorBot users. K: thousand.

During the data collection period (September 2018 to March 2019), 16,519 users interacted with DoctorBot to present health concerns. Our analysis of users’ demographic information allowed us to obtain an understanding of the user groups and their characteristics. As Figure 2 shows, more than one-half of the consultations (9052/16,519, 54.80%) were initiated by male users, and the mean ages of male and female users were 30 years and 27 years, respectively. In particular, the majority of DoctorBot users were between ages 20 and 39 years, which may be due to the relatively high technology proficiency and willingness to use advanced technologies (ie, intelligent chatbot) among this age group. Many users in the 20 to 39 years age group also consulted the application concerning their childrens’ illnesses (eg, “May I ask the reason why a 6-year-old girl often vomits?”). In contrast, only a small number of older adults (aged >60 years) or their caregivers (n=885) attempted to use the chatbot. This finding aligns with previous research showing that older adults are lagging in the adoption of novel health-related technologies [35]. To bridge the gap, more research is needed to examine how to design advanced health technologies (eg, chatbots) tailored to the aging population.

#### What Were the Length and Frequency of Chatbot Sessions?

**Figure 3:**
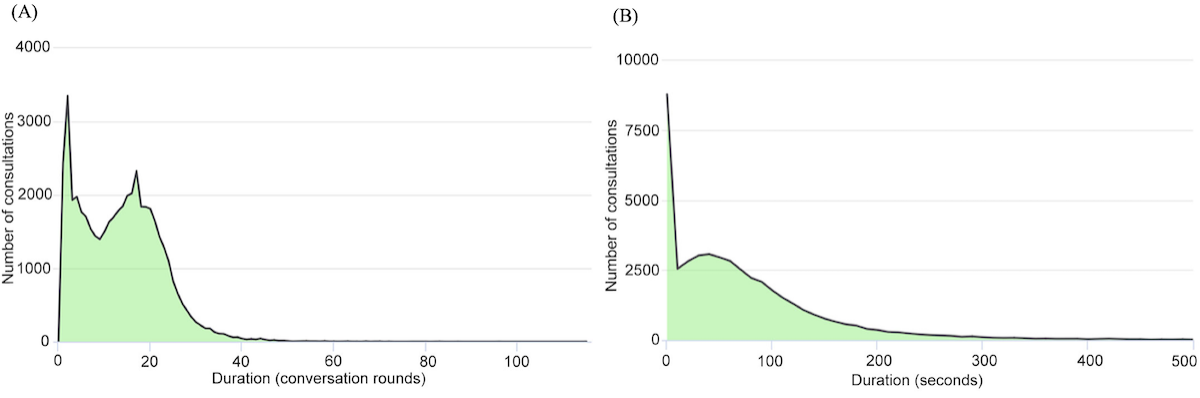
(A) Consultation duration in conversation rounds. (B) Consultation duration in seconds.

As shown in Figure 3, the duration of each consultation varied. Many consultation sessions only lasted a few conversation rounds or seconds, whereas some others took more than 20 conversation rounds or 5 minutes. This finding highlights that many users only interacted with the chatbot for a brief time, which may not have allowed them to complete a consultation session.

Regarding the frequency of chatbot use, we found that a large number of users (10,371/16,519, 62.78%) attempted to use the application only once. In some sessions, users presented more than one health issue and never used the application again. For those who used the chatbot more often (n=6148), some used the consultation service on one or two specific days. For example, one user who initiated 23 consultations with the chatbot only used the application on February 20, 2019, and March 13, 2019, to present two different health concerns (headache and palpitation). These findings suggest that even though DoctorBot attracted a lot of users, it was not used extensively. Furthermore, the significant number of “single-use” sessions reveals the issue of user engagement and retention; therefore, it is vital to understand what caused the low usage and how to optimize user experience to achieve the best uptake [23].

#### What Health Concerns Were Presented to DoctorBot?

**Figure 4:**
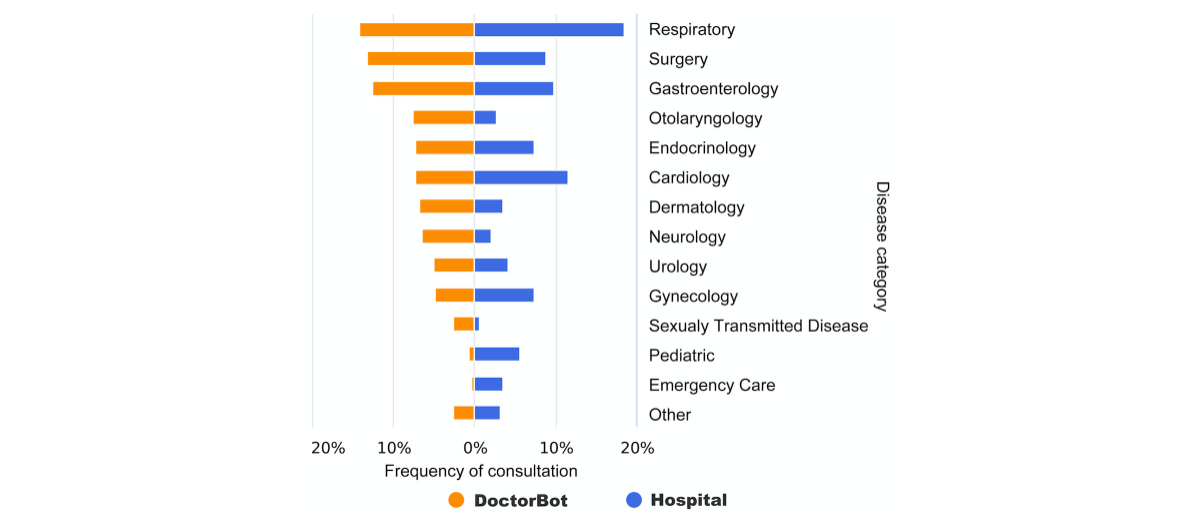
Comparison of the frequency of consultations according to the major disease category of the presenting illness between DoctorBot (orange bars) and primary hospitals (blue bars).

Each completed consultation session immediately returned a diagnostic report to the user. A typical report contains the major health concerns expressed by the user and their association with possible diseases that are automatically classified by DoctorBot based on a widely adopted disease classification schema [36]. We analyzed the generated diagnostic reports to understand what health concerns or diseases people usually presented to DoctorBot and the frequency with which each disease was presented. This step was followed by a comparison with the usage of health services in hospitals. In particular, we measured the frequency of consultation on DoctorBot for each disease type and compared that with the health service usage reported by three primary hospitals in China [37]. As Figure 4 shows, diseases with mild symptoms, such as those in the gastroenterology and dermatology categories, appeared in a lot of chatbot consultations, in proportions that were significantly higher than those seen in primary hospitals. One possible explanation is that people with mild symptoms would prefer using the chatbot to query the necessity of clinical visits first, rather than going to hospitals directly. In contrast, using DoctorBot to address emergency care issues was not very common. We also noticed the use of DoctorBot to seek help on medical conditions that often entail considerable privacy and social stigma issues, such as sexually transmitted diseases.

### Issues in the Use of DoctorBot

In this section, we report our analysis on the completed and uncompleted sessions, as well as on the user feedback, to present the major issues in the real-world use of DoctorBot.

#### Dropping Out of Consultations

**Figure 5:**
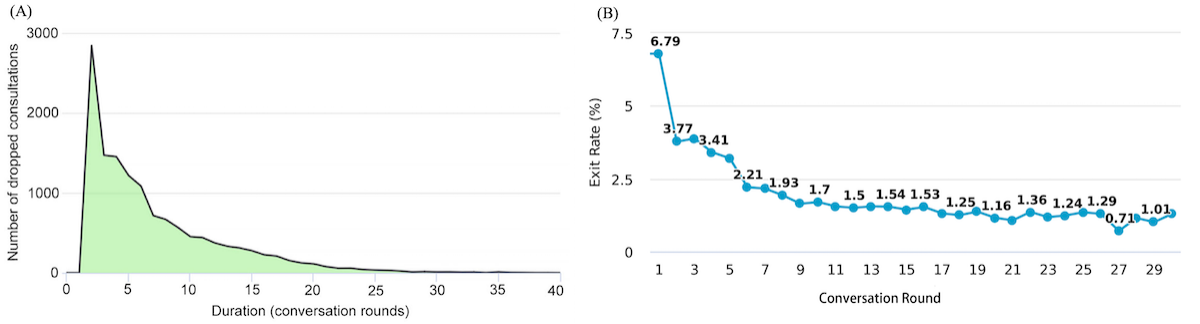
(A) Distribution of dropped consultation sessions over conversation rounds. (B) The exit rate for each conversation round.

Our data showed that there was a large number of uncompleted consultations, where users withdrew at a certain point without completing the process (16,974/47,684, 35.60%). In particular, more than one-half of the uncompleted consultations (9542/16,974, 56.22%) occurred within the first five conversation rounds (Figure 5A). To further verify our observation, we built a model to measure the exit rate for each conversation round:

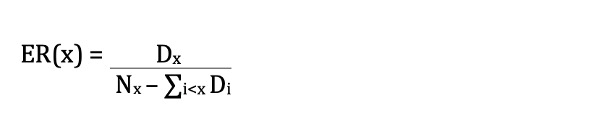



where ER(x) is the exit rate of dropping a conversation in round x, D_x_ is the number of conversations that drop in round x, and N_x_ is the number of all conversations.

As Figure 5B shows, the exit rate spiked at the beginning, signaling that a lot of users dropped out after a very brief interaction with the chatbot (ie, after just one round of conversation). Furthermore, the exit rate of the first five conservation rounds was much higher than for the rest. These findings are consistent with our observation that users most likely terminated the consultation at an early stage.

To further understand what caused user dropout, we examined the specific question asked by DoctorBot when a consultation was terminated and how much time users spent on answering those questions. As Table 1 demonstrates, transition questions, such as the ones prompting the user to answer the question again, often led to a termination of chatbot use. Furthermore, many uncompleted conversations occurred when users were asked to provide a detailed account of the symptoms they were experiencing. Our analysis also showed that such questions usually took longer than other relatively simple questions, such as questions about demographic information (91.1 s versus 17.6 s, respectively). One possible explanation is that symptom-related questions were usually hard to answer and could easily overwhelm or even confuse users, leaving them unsure of what input to provide and eventually causing them to terminate the conversation. For example, during a consultation for a headache, the chatbot asked whether the user was experiencing a series of symptoms related to headaches, such as fever, vomiting, stuffy nose, cough, and chest tightness. When the chatbot attempted to ask another follow-up question, the user suddenly terminated the consultation.

**Table 1:** The categories of questions asked by DoctorBot before users terminated the consultation.

Question category	Number of times users terminated their consultation when question was asked (%^a^)
Demographic information	58 (4.39)
Physiological data	208 (2.58)
Transition questions	5274 (14.74)
Symptoms	8469 (20.23)
Medical history	795 (4.26)

^a^Calculated as the number of times users terminated their consultation when a question was asked divided by the number of conversations that contained questions from that category.

**Figure 6:**
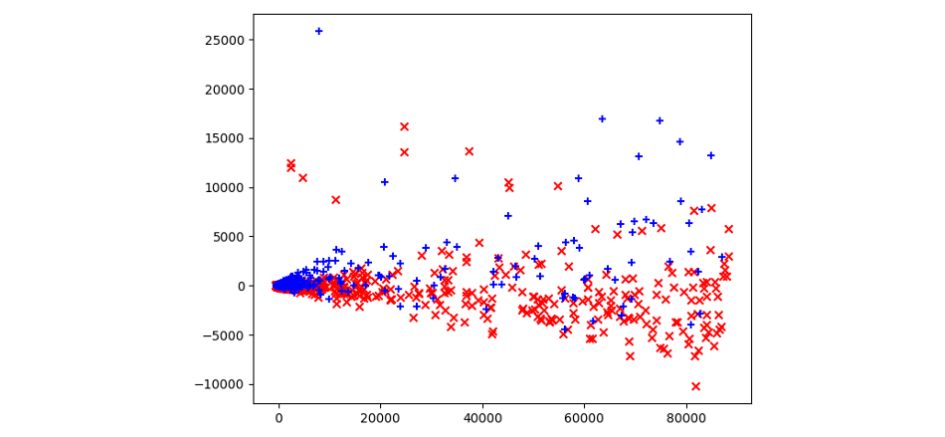
A principal component analysis (PCA) scatterplot of consultations for 30,710 completed (blue dots) and 16,974 dropped (red dots) consultations. PCA has successfully found linear combinations of the different features in a two-dimensional feature space that separate two different clusters corresponding to whether or not the consultations were completed.

Lastly, we analyzed the differences between completed and uncompleted sessions to identify the major characteristics of dropped consultations. In particular, we conducted PCA and built a binary logistic regression model using the following features: gender, age, duration of the consultation, number of conversation rounds, and average duration of each round. We included gender and age in the model because these two factors may influence users’ acceptance and use of technology [38,39]. As Figure 6 shows, despite that there were twice as many completed consultations as uncompleted consultations, most of the completed consultations (blue dots) were plotted in a small area in the two-dimensional feature space while the dropped consultations (red dots) were distributed horizontally, illustrating that considerable differences existed between completed and uncompleted consultations.

When we controlled the other features (eg, gender, age, and duration of consultation), logistic regression analysis showed that the number of conversation rounds (*P*<0.001) and the average duration of each round (*P*<0.001) significantly correlated with the dependent variable (whether the user dropped out in the middle of the consultation session) (Table 2). More specifically, as the number of conversation rounds with the chatbot increased, the likelihood of terminating the consultation decreased (odds ratio 0.7320, 95% CI 0.7298-0.7341). This finding is consistent with our observation that users most likely terminated the consultation at an early stage.

**Table 2:** The estimated coefficients, odds ratio, and *P* value of each feature in the logistic regression model with terminating consultation as the dependent variable.^a^

Feature	Estimated coefficients (SE)	Odds ratio (95% CI)	*P* value
Gender	3.19e–02 (2.95e–02)	1.0324 (1.0023-1.0633)	.28
Age	1.20e–03 (1.07e–03)	1.0012 (1.0001-1.0023)	.26
Duration of consultation	5.04e–05 (6.64e–06)	1.0001 (1.0000-1.0001)	<.001*
Number of conversation rounds	–3.12e–01 (2.99e–03)	0.7320 (0.7298-0.7341)	<.001*
Mean duration of each conversation round	–3.17e–04 (8.57e–05)	0.9997 (0.9996-0.9998)	<.001*

^a^McFadden pseudo *R*^2^=0.4338884.

*P<.001.

#### Using Chatbot for Nontherapeutic Purposes

**Figure 7:**
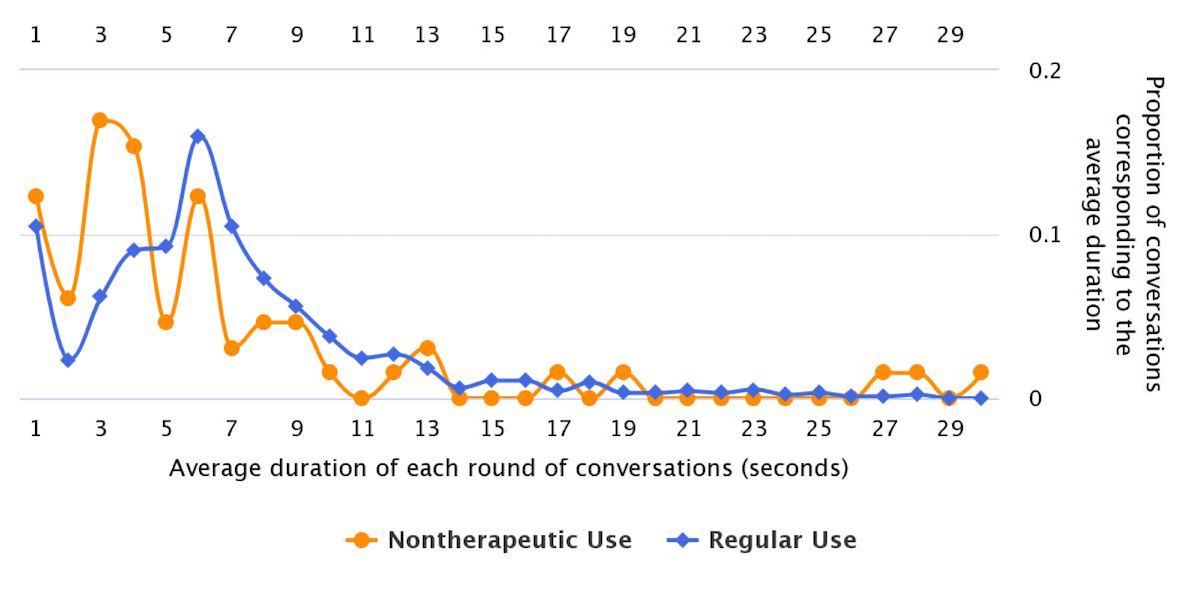
The distribution of the average duration of conversations.

Through the analysis of 3000 randomly selected completed consultation sessions, we noticed another issue in the use of DoctorBot: users often pretended to have health concerns and did not always use the chatbot for therapeutic purposes (ie, medical consultations). In fact, they “gamed” the chatbot, a user behavior that has been reported in intelligent tutoring systems [40] and workplace chatbot systems [41]. This behavior was exhibited in 241 of 3000 (8.03%) consultation sessions. We characterized the “nontherapeutic” use of DoctorBot into 5 patterns (Table 3). For example, people used nonsense/illogical words (eg, “I miss you”) or contradictory statements to describe their health concerns. They might have even used the same word (eg, “nothing,” “unclear”) throughout the entire consultation. We also compared regular and nontherapeutic consultation sessions in terms of the mean duration of conversations. It was shown that the proportion of nontherapeutic and regular sessions with a conversation round lasting up to 4 s was 50.76% and 17.53%, respectively (Figure 7). This finding revealed that users who used the chatbot for nontherapeutic purposes attempted to complete each round of conversation as quickly as possible. We also analyzed the standard deviation of time taken per conversation round, which showed that users with real health concerns spent various lengths of time on different questions (mean 11.85 s, SD 17.06 s). In contrast, users who didn’t actually use the chatbot for medical consultation spent an equally short time on each question (mean 7.74 s, SD 14.33 s).

**Table 3:** Patterns of nontherapeutic chatbot use and illustrative examples.

Patterns	Example
Extensive use of nonsense/illogical words	Chabot: If you have any other symptoms, please continue typing. User: I miss you.
Contradictory statements	Chatbot: Please tell me your symptoms. User: How can I deal with a headache? Chatbot: Which of the following symptoms does your description fit? User: Fever. Chatbot: What is your highest temperature? User: Unknown. Chatbot: How about the degree of headache? User: Painless.
Continuously use the same word to answer all the questions	Chatbot: Please tell me your symptoms. User: Headache. Chatbot: What kind of headache is it? User: Unclear. Chatbot: Do you have dizziness? User: Unclear. Chatbot: Is it accompanied by elevated blood pressure, chills, and other symptoms? User: Unclear. Chatbot: Is it accompanied by neck pain? User: Unclear. […]
Answering each question quickly	In the previous example, the user went through 19 rounds and spent 49 seconds, averaging only 2.6 seconds per question.
Spending an equally short time on each question	In the previous example, the user answered almost all of the questions in 2-3 seconds, and the SD of time was 1.2 seconds.

**Figure 8:**
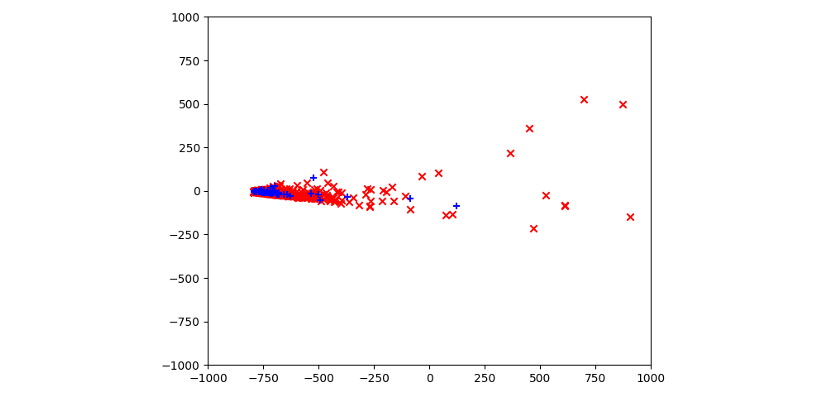
A principal component analysis (PCA) scatterplot of consultations for 2759 regular (blue dots) and 241 nontherapeutic (red dots) consultations. PCA has successfully found linear combinations of the different features in a two-dimensional feature space that separates two different clusters corresponding to whether or not the chatbot was used for a therapeutic purpose.

To further verify our findings of the patterns of nontherapeutic chatbot use, we conducted PCA and built a binary logistic regression model using the following features: gender, age, duration of a consultation, number of conversation rounds, mean duration of each round, time spent in each round, and number of conversation rounds with continuous repetition of the same response. We did not consider contradictory statements or extensive use of nonsense/illogical words in the analysis because it was not appropriate to quantify them. As shown in Figure 8, the regular consultations (blue dots) are plotted in a small area (the number of blue dots is 10 times greater than the number of red dots), whereas the nontherapeutic use cases (red dots) are scattered. This shows that there were obvious abnormalities in many nontherapeutic use cases.

Our logistic regression analysis showed that, when controlling other features, the mean duration of each round (*P*=0.03), the standard deviation of time spend in each round (*P*=0.05), and repetition of the response (*P*<0.001) significantly correlated with the occurrence of nontherapeutic chatbot use (Table 4). This suggests that for a consultation session, the shorter the average duration of each round was, the more likely the user was gaming the chatbot (odds ratio 0.9618, 95% CI 0.9409-0.9892). Also, the result suggests that if the duration of different conversation rounds was more varied, users were less likely to be using the chatbot for a nontherapeutic purpose (odds ratio 0.9835, 95% CI 0.9733-0.9939).

**Table 4:** The estimated coefficients, odds ratio, and *P* value of each feature in the logistic regression model, with using the chatbot for nontherapeutic purposes as the dependent variable.a

Feature	Estimated coefficients (SE)	Odds ratio (95% CI)	*P* value
Gender	6.28e–02 (2.98e–02)	1.0648 (1.0335-1.0970)	.09
Age	–1.66e–02 (1.05e–02)	0.9835 (0.9733-0.9939)	.11
Duration of consultation	–3.31e–03 (1.81e–03)	0.9967 (0.9949-0.9985)	.07
Number of conversation rounds	–4.60e–02 (3.17e–02)	0.9550 (0.9252-0.9858)	.15
Average duration of each round	–3.89e–02 (2.20e–02)	0.9618 (0.9409-0.9892)	.03*
SD of time spent in each round	–2.31e–02 (1.22e–02)	0.9772 (0.9653-0.9892)	.05*
Rounds of continuous repetition of the same response (unclear or unknown)	4.56e–01 (6.24e–02)	1.5778 (1.4823-1.6793)	<.001**

^a^McFadden pseudo *R*^2^=0.2478434.

*P<.05.

**P<.001.

#### User Concerns

**Figure 9:**
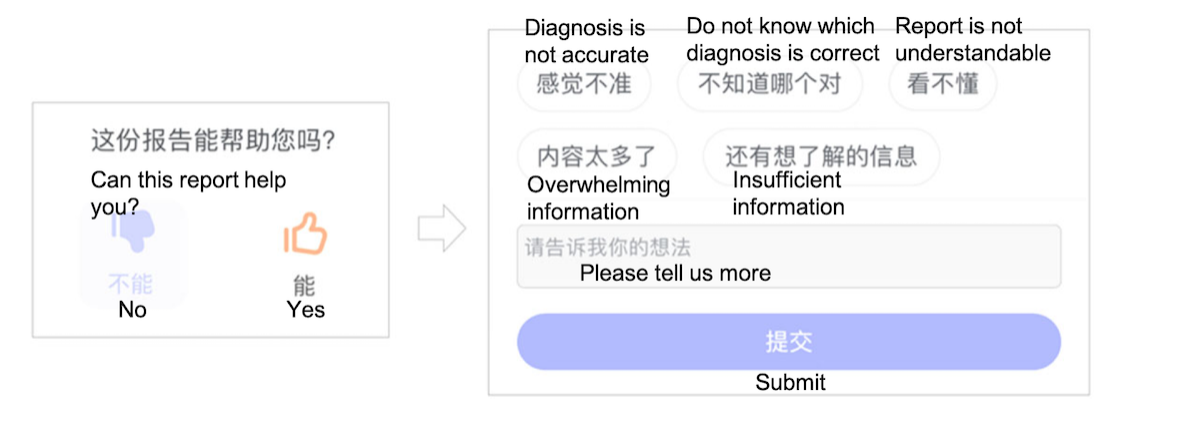
Interface for collecting user feedback: rating (left), predefined reasons (top right), and comment box (bottom right).

Toward the end of each consultation, DoctorBot prompted the user to rate the experience as either positive or negative (Figure 9). If a negative rating was chosen, the system asked the user to provide further feedback, which users could choose to do by either typing into a comment box or selecting from a list of predefined reasons (eg, “diagnosis is not accurate” or “overwhelming information”). Despite this step being optional, many users provided feedback. In total, we collected 3832 pieces of user feedback, with 2172 positive ratings and 1660 negative ratings. As negative ratings usually suggest that users had concerns, we examined the specific reasons for negative ratings by analyzing the textual feedback and the predefined reasons selected by users.

We characterized five user concerns that led to negative ratings (Table 5). We found that users usually expressed dissatisfaction when they believed that the diagnostic suggestion was not accurate; as one user stated, “I’m 21 years old with a regular lifestyle. I don’t smoke or drink. I eat and exercise regularly. You told me I have diabetes!!!” Some users even compared the diagnostic suggestions of DoctorBot with their physician’s diagnosis and stated that there was a discrepancy: “My doctor told me there is nothing to worry about and prescribed me an herbal medication. But the chatbot suggested a different diagnosis and I don’t trust it.” Furthermore, if users had difficulty comprehending the diagnostic report generated by DoctorBot or determining which diagnostic suggestion was more reliable, users also gave negative ratings. For example, one user commented, “I have no clue about the suggested diagnosis. What is that?” In other cases, users complained that the provided information on the diagnostic report was overwhelming. Indeed, due to different levels of health literacy, knowledge, and experience, users may have had challenges in comprehending the technical aspects of the diagnostic report (eg, medical jargon and professional medical knowledge) [42-44]. Lastly, users tended to give negative ratings if their information needs were not fully met. The analysis of textual feedback revealed that users desired to receive more personalized and actionable information, including medical information related to their health concerns, where to seek medical help, what to do next, and detailed explanations about the suggested diagnoses.

**Table 5:** Reasons cited by DoctorBot users for giving a negative rating of their experience.

User concern	Number of users
Suggested diagnosis is perceived to be inaccurate	1084
Difficult to assess, which suggested diagnosis is correct	164
Insufficient information	247
Report is not easy to understand	113
Provided information is overwhelming	52

**Figure 10:**
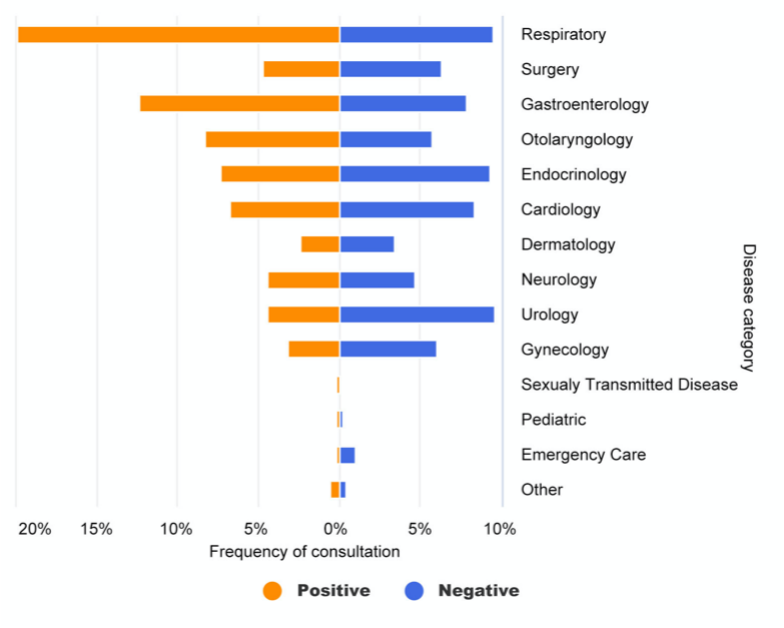
Relationship between user ratings and disease category of presenting illness of DoctorBot consultations.

We also analyzed the relationship between negative ratings and several intrinsic factors (eg, age, gender, duration of consultation, and disease type) to further examine if the user experience was affected by other factors that were not revealed by the analysis of user feedback. In particular, we found that the total time spent on the consultation had a significant impact on user experience. If a consultation lasted more than 2 minutes, users tended to rate their experience as negative. Interestingly, the disease type was also highly related to users’ experiences and their satisfaction level (Figure 10). For example, medical advice about common diseases, such as respiratory issues, usually received positive ratings. One possible explanation is that the chatbot could easily diagnose these diseases and provide pertinent information and medical advice to fulfill the users’ needs. However, it is challenging for the chatbot to provide an accurate diagnosis of and meaningful information about diseases with complex causal mechanisms, such as gynecopathy, based on only a few rounds of conversation; as such, negative ratings against DoctorBot-generated outputs were fairly common under such circumstances.

## Discussion

### Principal Findings

The scarceness and imbalanced distribution of health care resources (eg, facilities and doctors) are major health concerns worldwide [7,8]. Many people, especially those in rural areas, may not have immediate and convenient access to the medical services they need. Furthermore, due to the complex organization and workflow of conventional health care services, it is challenging for patients and caregivers to navigate the health care system [45]. As such, more digital tools have been introduced to help patients triage before seeing a doctor and gain information to fill their knowledge gap. For example, as predecessors of self-diagnosis chatbots, online symptom checkers have been launched to more effectively provide possible alternative diagnoses to patients and direct them to the appropriate care settings [46]. Prior work illustrated that symptom checkers have an acceptable level of patient compliance with medical advice [47] and triage accuracy [48]. However, symptom checkers often use some proprietary diagnostic algorithms, such as branching logic and Bayesian inference, that are not optimal for processing complicated and sometimes ambiguous user inputs, compromising not only user experience but also diagnostic accuracy [47,49].

With the recent advent of AI technologies such as knowledge graphs and deep neural networks, more sophisticated self-diagnosis health chatbots are emerging to simulate the conversations between patient and care providers and provide more accurate and comprehensive care advice and services. The application of chatbot technology in the health domain, including mental health [21] and behavioral therapy [50,51], is becoming more and more common. Despite the effectiveness of chatbots in delivering health care services to improve well-being, this novel technology has not been adopted at the rates predicted based on the high level of interest [25]. Due to the lack of large-scale deployment, prior work primarily examined the use of health chatbots in controlled settings rather than in a real-world context [28]. This study aimed to bridge the knowledge gap in how people use health chatbots in real-world scenarios.

To that end, we analyzed the system log of a self-diagnosis chatbot to understand how health care chatbots would be used in the real world and what issues could impede the optimal use of this novel technology. We found that users in all age ranges, including middle-aged and older adults, had used the chatbot. A considerable number of people used the chatbot only once. Users consulted the chatbot about a wide range of topics, including mild medical conditions, as well as those that often entail considerable privacy and social stigma issues. We also observed several issues in the use of the chatbot, including user dropout and use for nontherapeutic purposes. Finally, we identified a set of user concerns that should be addressed to optimize user experience, including receiving insufficient actionable information and perceived inaccurate diagnostic suggestions. We argue that designers and developers of health chatbots need to employ user-centered approaches to address users’ concerns and issues. Below we discuss design implications for health chatbots to enhance user experience and engagement.

### Design Implications

#### Designing Informative Health Chatbots

The analysis of user feedback revealed that users expressed the need to receive more actionable information, such as next steps to take. This suggests that users’ information needs were not adequately met by the chatbot. Also, users complained that the system-generated diagnostic report was difficult to interpret. These findings highlight the importance of providing more useful information that patients need. For example, in the diagnostic report, chatbots could provide links to consumer-friendly and credible information sources to help patients better understand the content of the report [42].

#### Designing Easy-To-Use Health Chatbots

Chatbots typically ask consecutive questions about concomitant symptoms so that they can generate a more accurate diagnosis. However, these questions are usually hard to answer and can easily overwhelm the user. For example, we found that users tended to terminate the consultation when they were asked to describe their symptoms or chief complaints. To address these issues, it would be useful to allow users to share and describe information in the form of voice recordings to reduce the amount of time and effort spent on typing. The chatbots should also be designed to inform users as to why a particular piece of information is needed [52].

Another interesting finding is that a small number of elderly people also used the application. Given the rise in the aging population and its associated health care costs in many countries, health chatbots will likely become a promising approach to aid older adults’ independent living [53]. This raises a new set of questions for chatbot designers about how to make health chatbots more accessible and user-friendly for older adults. For example, a speech interface can be incorporated into chatbot systems to facilitate communication between elderly users and health chatbots [54].

#### Designing Trustworthy Health Chatbots

Through the analysis of user feedback, we found that perceiving the chatbot’s output (eg, recommended diagnosis) as inaccurate was highly associated with a negative user experience. This is not surprising because health care has a high degree of criticality and users tend to have doubts about the diagnosis suggested by chatbots. Therefore, to better engage users, it is very important to increase the trustworthiness of health chatbots. Prior literature on AI-driven intelligent systems has suggested presenting system outputs in a format that is meaningful, understandable, and trustworthy to help users better understand the system’s hidden intelligence and then determine whether it is appropriate to trust the recommendations and use them to make decisions [55]. In particular, prior work suggested presenting a variety of system-related information to the user, including system reliability and performance data, logic, and reasoning (eg, how the system operates and how its outputs are generated), as well as the information sources that the system leverages to produce the output [52,56,57]. Aligning with those arguments, we suggest that health chatbots need to explain data sources, prediction accuracy, and how the diagnostic report is generated to the users to build trust. For example, chatbots could provide more appropriate explanations to indicate what types of diseases they are knowledgeable of and their degree of confidence in their diagnostic suggestion.

#### Designing Onboarding Experiences for Users

We observed that many users dropped out of consultations, especially during their early phases. This finding highlights the necessity of enhancing user engagement at an early point. Furthermore, the chatbot was sometimes used for nontherapeutic purposes. We speculate that because self-diagnosis chatbots are an emerging technology, some users may just want to navigate through the application to explore how the chatbot works. However, gaming the chatbot could generate a large amount of noisy data, some of which might be used to train models; therefore, nontherapeutic use cases, if not taken care of properly, could adversely affect the performance of health chatbots. To prevent these issues and better engage users, it may be useful to provide them with onboarding materials during the initial interactions. Prior work has suggested that onboarding materials could educate users about the most effective way to use advanced technologies (ie, AI-driven health chatbots) [58]. As an example, the onboarding materials could introduce users to the basic functions (eg, capabilities and limitations) of the chatbot and the process of consultation (eg, what types of questions will be asked and why). Moreover, the chatbot should be designed to automatically detect and tag nontherapeutic use cases so that developers could easily remove such noisy data when training AI models.

### Limitations

The present study has several limitations. First, this study relied heavily on log data. Even though the analysis of log data could provide valuable insights into the use of the chatbot in real-world settings, it didn’t allow us to capture the perceptions and opinions of users when interacting with the chatbot, such as what features they liked and disliked, what barriers they encountered, and how the chatbot should be improved to optimize the user experience. In the future, we will employ social theories on the explanatory factors of the use of technology (eg, diffusion of innovation) to conduct user studies (eg, interview, survey, and usability evaluations) with different groups of people to form a more comprehensive view of users’ attitudes and experiences about this novel technology. Second, we only examined the use of one health chatbot, which is likely to compromise the generalizability of the findings. To assess and expand our results’ generalizability, it would be useful to examine other chatbots, including specialist chatbots that serve a particular population with specific conditions. Lastly, cultural and social factors could also play a vital role in the utilization of health chatbots. We will also examine those issues in our future work.

### Conclusions

In this paper, we conducted both quantitative and qualitative analysis on the system log of a self-diagnosis chatbot, which has been widely deployed in China. To the best of our knowledge, this is the first study examining the use of health chatbots in the real world using a large-scale, heterogeneous data set. We described our general observations of the chatbot’s use, including who used it, how long and how often they used the application, and what health concerns were presented. Furthermore, we analyzed both completed and uncompleted consultation sessions as well as the user feedback to investigate issues that may hinder the effective use of the chatbot. These results shed light on the design of health chatbots to improve user experience and increase user engagement.

## Abbreviations

AI: artificial intelligence
mHealth: mobile health
PCA: principal component analysis
